# *Penicillium chrysogenum* polypeptide extract protects tobacco plants from tobacco mosaic virus infection through modulation of ABA biosynthesis and callose priming

**DOI:** 10.1093/jxb/erab102

**Published:** 2021-03-03

**Authors:** Yu Li, Mengting Jiao, Yingjuan Li, Yu Zhong, Xiaoqin Li, Zhuangzhuang Chen, Suiyun Chen, Jianguang Wang

**Affiliations:** 1 Biocontrol Engineering Research Center of Crop Disease & Pest of Yunnan Province, School of Life Science, Yunnan University, Kunming, China; 2 Biocontrol Engineering Research Center of Plant Disease & Pest, School of Life Science, Yunnan University, Kunming, China; 3 University of Antwerp, Belgium

**Keywords:** ABA, callose, infection, *N. benthamiana*, plasmodesmata, PDMP, priming, tobacco mosaic virus (TMV)

## Abstract

The polypeptide extract of the dry mycelium of *Penicillium chrysogenum* (PDMP) can protect tobacco plants from tobacco mosaic virus (TMV), although the mechanism underlying PDMP-mediated TMV resistance remains unknown. In our study, we analysed a potential mechanism via RNA sequencing (RNA-seq) and found that the abscisic acid (ABA) biosynthetic pathway and β-1,3-glucanase, a callose-degrading enzyme, might play an important role in PDMP-induced priming of resistance to TMV. To test our hypothesis, we successfully generated a *Nicotiana benthamiana* ABA biosynthesis mutant and evaluated the role of the ABA pathway in PDMP-induced callose deposition during resistance to TMV infection. Our results suggested that PDMP can induce callose priming to defend against TMV movement. PDMP inhibited TMV movement by increasing callose deposition around plasmodesmata, but this phenomenon did not occur in the ABA biosynthesis mutant; moreover, these effects of PDMP on callose deposition could be rescued by treatment with exogenous ABA. Our results suggested that callose deposition around plasmodesmata in wild-type plants is mainly responsible for the restriction of TMV movement during the PDMP-induced defensive response to TMV infection, and that ABA biosynthesis apparently plays a crucial role in PDMP-induced callose priming for enhancing defence against TMV.

## Teaser

Fungal polypeptide-induced callose priming restricts the cell-to-cell movement of tobacco mosaic virus in *N. benthamiana* via the ABA biosynthetic pathway.

## Introduction

Plants are unique organisms because they are sessile and cannot escape potential aggression and threats posed by some biotic or abiotic factors. Some pathogens (biotic factors) that successfully invade plants can overcome or evade host immune defences; these pathogens can cause plant diseases and thus threaten plant survival and agricultural production. Viruses are composed of nucleic acid molecules and proteins and can proliferate only in living cells. Throughout the long-term struggle between plants and viruses, plants have evolved a variety of mechanisms to resist viruses. In general, the plant immune system consists of two levels of immune responses: (i) the first involves the ability of plants to recognize pathogen-associated molecules (pathogen-associated molecular patterns, PAMPs) via transmembrane receptors on the surface of plant cells, and this recognition process is referred to as PAMP-triggered immunity (PTI); and (ii) the second involves the ability of resistance genes (*R* genes) that encode intracellular receptors in plants, to specifically recognize pathogen effectors to activate effector-triggered immunity (Jones and Dangl, 2006; Schwessinger and Ronald, 2012).

Many recent studies have reported a potentiated defence capability that is not accompanied by the induction of specific defence genes until the plant is infected; this phenomenon is referred to as ‘priming’ (Katz *et al.*, 1998; Conrath *et al.*, 2002; 2006; Gamir *et al.*, 2014; Crisp *et al.*, 2016; Mauch-Mani *et al.*, 2017). In one of the earliest studies on seed pre-treatment techniques, Heydecker *et al.* (1973) proposed the word ‘priming’ as a treatment technique that allows for slow water absorption and gradual seed drying; this technique is also known as seed osmotic regulation. When a plant or part of a plant senses a warning signal from biotic or abiotic factors, it induces a preparatory defensive response. This plant priming process is a type of physiological readiness that enhances defences when plants are re-damaged by insects or microorganisms; this process accelerates and strengthens the plant defence responses (Conrath *et al.*, 2006; Beckers and Conrath, 2007; Kim and Felton, 2013). Priming is believed to be an intrinsic part of induced resistance. Notably, priming protects against pathogens as well as insect arthropods (Engelberth *et al.*, 2004; Frost *et al.*, 2008) and abiotic stress (Beckers and Conrath, 2007; Theocharis *et al.*, 2012).

Elicitors can induce a range of defence responses in plants, including a burst of reactive oxygen species, secondary metabolite accumulation, and pathogen-associated protein expression (Wojtaszek, 1997; van Loon *et al.*, 2006). Dry mycelium of *Penicillium chrysogenum* (DMP), which constitutes the residual by-product of industry-produced penicillin, does not contain penicillin or live mycelium. It was first reported that extracts of dead *P. chrysogenum* can induce resistance against *Fusarium* wilt in melon (Dong and Cohen, 2001). It was then reported that DMP can protect cucumber and tomato plants against the root-knot nematode *Meloidogyne javanica* (Gotlieb *et al.*, 2003). Subsequent studies have shown that an aqueous extract of DMP induces resistance in several crop species under both controlled and field conditions (Thuerig *et al.*, 2006). DMP has been widely used in flue-cured tobacco planting in Yunnan province, China, and the results have yielded good economic and ecological benefits (Yang *et al.*, 2013). However, current studies on DMP have focused mainly on its regulatory effects on crop species. The plant defence response and disease resistance mechanism induced by DMP should be systematically studied to lay a foundation for widespread applications of DMP in crop production. However, the mechanisms through which DMP induces resistance to tobacco mosaic virus (TMV) remain unclear, and related studies are scarce. A polysaccharide (PCPS) and polypeptide extract (PDMP) of DMP have been successfully isolated in our laboratory (Fu *et al.*, 2020). Our previous study indicated that polysaccharides of DMP induced nitric oxide (NO) and hydrogen peroxide (H_2_O_2_) production to initiate an early defence response, which in turn improved the resistance of *Nicotiana glutinosa* to TMV (Fu *et al.*, 2020). However, the effect and underlying mechanism of PDMP on tobacco plants remain unknown. In this study, we used PDMP to investigate the underlying mechanism of its antiviral effect on *Nicotiana benthamiana*. We speculated that PDMP can be used as a disease resistance inducer to prevent disease.

Abscisic acid (ABA) is a hormone that regulates plant growth and development. ABA is a type of vascular plant hormone that was named for its ability to promote abscission (leaf loss), and is abundant in the organs and tissues of higher plants, particularly in mature and senescent tissues and in organs beginning to senesce. In higher plants, ABA is synthesized through the carotenoid pathway. In the initial reaction, carotene from β-carotene precursors serves as a precursor of intermediate reactions in plastids. In the following reaction, zeaxanthin in plastids is lysed to form xanthoxin through reactions catalysed by members of the 9-*cis*-epoxycarotenoid dioxygenase (NCED) family. In the last reaction, xanthoxin is converted to ABA, which is catalysed by xanthoxin dehydrogenase (ABA2) and aldehyde oxidase (AO3; Seo and Koshiba, 2002; Nambara and Marion-Poll, 2005; Wasilewska *et al.*, 2008). When plants are dehydrated, the ABA concentration sharply increases, and drought stress promotes the widespread biosynthesis of ABA in the roots, xylem, and leaves (Davies and Zhang, 1991). Exposure to water-restriction stress, which leads to dehydration, induces the expression of zeaxanthin epoxidase (*ZEP*) in the roots (Audran *et al.*, 1998; Thompson *et al.*, 2000). Under drought conditions, the expression of *ZEP* in the roots plays an important role in controlling the biosynthesis of ABA. Under normal water conditions, plants produce endogenous amounts of ABA; moreover, the amount of ABA in a tobacco *aba2* mutant could be compensated by overexpression of the *ZEP* gene (Borel *et al.*, 2001). Thus, in our study, using clustered regularly interspaced short palindromic repeats (CRISPR)/CRISPR-associated protein 9 (Cas9) technology, we blocked the ABA biosynthetic pathway by mutating the *ZEP* gene. With an editing efficiency of 85.4%, the powerful CRISPR/Cas9 gene-editing vector system allows for easy, rapid genome editing of multiple monocot and dicot species (Ma *et al.*, 2015; Mao *et al.*, 2019). Although many studies have been performed to improve and optimize the CRISPR technique, off-target events cannot be completely avoided (Liu *et al.*, 2017). To determine the mutation events of a target, a classic decoding method is used, which involves amplifying the DNA fragments containing the target sequence by using special primers, constructing clones, and selecting several clones for Sanger sequencing. In this study, we tested not only T_0_ plants but also T_1_ and T_2_ plants, and selected mutants that were stable in the T_0_, T_1_, and T_2_ generations for subsequent experiments.

Callose is a β-1,3-glucan cell wall polymer (Xie and Hong, 2011) that can be reversibly deposited around the neck of plasmodesmata when a plant is injured (Radford *et al.*, 1998; Roberts and Oparka, 2003), and this deposition has a certain regulatory effect on the permeability of plasmodesmata (Northcote *et al.*, 1989; Delmer *et al.*, 1993). Therefore, callose is believed to act as a physical barrier that limits or prevents virus transport between cells. Callose deposition around plasmodesmata is the result of a dynamic regulatory process that involves stimulation of β-1,3-glucanase and callose synthase. Callose usually accumulates in the cell wall around both ends of the plasmodesmata, and this accumulation presses the plasma membrane inward to produce a narrow neck (Hughes and Gunning, 1980; Ding *et al.*, 1992; Radford *et al.*, 1998), thus reducing the space available for the transport of materials through the plasmodesmata (Takahashi and Jaffe, 1984; Northcote *et al.*, 1989).

In this study, we first analysed the effects of PDMP on TMV infection via RNA-seq. We also successfully generated a *N. benthamiana ZEP* mutant, and investigated the potential mechanism involved in PDMP-mediated resistance to TMV infection. Our study provides new insights into the molecular mechanism of PDMP-mediated resistance to TMV infection in tobacco.

## Materials and methods

### Plant material and treatment

Seeds of wild-type *N. benthamiana* were obtained from the seed stock bank of our laboratory (Center for Plant Disease and Pest Biocontrol, Kunming, China). The plants were grown in a greenhouse under a day/night temperature of 28/23 °C, humidity of 50–80%, and light/dark photoperiod of 14 h/10 h. An *Agrobacterium* strain [Agro-p35s-m30B: green fluorescent protein (GFP)] harbouring TMV was donated by Professor Xiaoying Chen from the Institute of Microbiology, Chinese Academy of Science. The leaves of the plants in the PDMP treatment were uniformly smeared with 1 mg ml^-1^ PDMP solution, and those in the TMV treatment were agroinfiltrated with TMV via a 1 ml syringe without a needle. Infiltration buffer containing TMV was prepared as described by Zhong *et al.* (2015). We adopted the method of local injection of leaves. First, a sterile 1 ml needle was used to gently prick a needle eye on the surface of the leaves, paying attention not to pierce the leaves. With the index finger against the needle eye position, the other hand slowly injected the infiltration buffer (about 100 µl ) into the leaf from the needle eye using a 1 ml syringe without a needle. The leaves of the plants in the PDMP/TMV treatment were pre-treated with PDMP for 7 d and then inoculated with TMV, and the leaves of the plants in the control treatment were smeared with distilled water only. For ABA treatment, an ABA solution (100 µ M; A8060, Solarbio, Beijing, China) was sprayed onto the leaves one day before inoculation with TMV, and after that, the leaves were sprayed with ABA solution every 3 d until the materials were collected.

### RNA extraction and transcriptome analysis via RNA-seq

For RNA-seq, leaves were harvested at 7 d post-inoculation (dpi), combined into one composite sample, and then flash-frozen in liquid nitrogen. Each treatment consisted of three biological replicates. Total RNA was extracted using TRIzol reagent (Takara, Tokyo, Japan) according to the manufacturer’s instructions. The RNA quality and purity were assessed using NanoDrop 2000 (Thermo, Waltham, USA) and an Agilent 2100 (Agilent Technologies, CA, USA) system based on an RNA integrity number (RIN) of at least 8.0. Transcriptome sequencing was performed using the high-throughput Illumina HiSeq 4000 sequencing platform (Illumina, San Diego, CA, USA).

### RNA-seq data analysis

The reference genome used to analyse our RNA-seq data was version Niben101, and was obtained from ftp://ftp.solgenomics.net/genomes/Nicotiana_benthamiana/. Based on the selected reference genome sequence, the mapped reads were combined via StringTie or Cufflinks software. The mapped reads were compared with the original genome annotation information to identify the original unannotated transcription region and to discover new transcripts and new genes of the species to supplement and improve the original genome annotation information. The differentially expressed genes (DEGs) were identified based on their fragments per kilobase per million reads (FPKM) via RSEM (RNA-Seq by Expectation-Maximization; Krylov *et al.*, 2003). DEGs with an adjusted *P* value ≤0.05 and log_2_(fold change) (log_2_FC) ≥1 were used to identify the number of DEGs in the different treatments. DESeq2 was subsequently used to determine the false discovery rate (FDR) threshold (adjusted *P* value). The FDR in the multigroup comparison was less than 0.05, and this value was considered to indicate significantly different expression.

### Quantitative real-time polymerase chain reaction (qRT–PCR)

Total RNA was obtained from leaves using TRIzol reagent (Takara, Tokyo, Japan). The RNA was then reverse transcribed into cDNA using PrimeScript™ RT Master Mix (Takara, Tokyo, Japan). The cDNA content was subsequently measured via an ABI 7500 system (Thermo, Waltham, MA, USA) in conjunction with SYBR™ Green PCR Master Mix (Thermo, Waltham, MA, USA). The cycle threshold (CT) values of the target genes were normalized to the CT value of *β-actin*/*EF1α*/*F*-*box*. The PCR conditions were as follows: 95 °C for 5 min, followed by 40 cycles of 95 °C for 15 s and 60 °C for 30 s. The relative expression was analysed according to the 2^−ΔΔCt^ method. The sequence data for *ABA*2 (EU123520.1), *ZEP* (XM_016582459.1), *β -actin* (AB158612) and β *-1,3-glucanase* (Niben101Scf01001g00005) were retrieved from the NCBI data libraries. The relative expression of the target genes was normalized to those of multiple endogenous control genes, such as the β *-actin*, *EF1α,* and *F-box* genes (Nakano and Mukaihara, 2019). The primers used for qRT–PCR are listed in Supplementary Table S1.

### TMV visualization

An *Agrobacterium* strain [Agro-p35s-m30B: green fluorescent protein (GFP)] harbouring TMV was donated by Professor Xiaoying Chen from the Institute of Microbiology, Chinese Academy of Science. The GFP fluorescence of the TMV-inoculated plants was observed under a handheld UV lamp (365 nm; Model sb-100 p/FA, Spscientific, USA), and the plants were imaged with a digital camera (Canon 500D, Canon, Japan). The GFP fluorescence of the plants was quantified using ImageJ 2x software.

### CRISPR/Cas9

CRISPR/Cas9 pP1C vectors were purchased from Genloci Biotechnologies Inc. (Cat. No. GP0143, Nanjing, China). The experiments were performed according to the manufacturer’s instructions. The sequence of the target for CRISPR/Cas9-induced mutations of *N. benthamiana ZEP* is ‘GCTGCCTTTATTGATCTCTAAGG’. *ZEP* from mutations was amplified and sequenced for verification using the forward primer 5′-ATGTATTCAACTGTGTTTTACACTT-3′ and reverse primer 5′-ACCAGTTACCAGAAACACCATCAAC-3′.

### Plant transformation and mutation analysis

pP1C.4 vectors containing gRNA and Cas9 expression cassettes were transformed into *Agrobacterium tumefaciens* GV3101 according to the freeze-thaw method. The positive clones were then used to produce *NbZEP* mutant *N. benthamiana* via the *Agrobacterium*-mediated leaf disc transformation technique (Pathi *et al.*, 2013; Hudzieczek *et al.*, 2019). Hygromycin-resistant seedlings were obtained, and the mutants were subsequently identified using PCR amplification and Sanger sequencing. gDNA was extracted from the T_0_ transgenic plants via a MiniBEST Plant Genomic DNA Extraction Kit (Takara, Tokyo, Japan). For the detection of mutations, the genomic regions that included the Cas9/gRNA target sites were amplified via PCR in conjunction with specific primers (see CRISPR/Cas 9 methods). The PCR products were then cloned into T vectors by using a pEASY-T5 Zero Cloning Kit (TransGen, Beijing, China), and the positive clones were sequenced via Sanger sequencing. The resulting reads were aligned with wild-type sequences for the detection of candidate mutant lines using DNAMAN 8.0 software (Lynnon Biosoft Company, USA). To investigate the inheritance of CRISPR/Cas9-induced targeted *NbZEP* modifications in subsequent generations, three T_0_ plants with biallelic mutations were self-pollinated, and the resulting T_1_ plants were then transplanted into soil and grown to maturity. Mutant tobacco lines were identified genetically according to the above-described method. The mutant T_2_ plants were then used for further analysis.

### Quantification of ABA using ELISA

Leaves of wild-type *N. benthamiana* and the *ZEP* mutant were collected. The ABA concentrations in the leaf tissue homogenates were measured with a plant ABA ELISA kit (Mlbio, Shanghai, China), and the experiment was performed according to the manufacturer’s instructions. The optical density (OD) value of the samples at 450 nm was measured with a microplate reader (SpectraMax iD5, Molecular Devices, USA).

### Aniline blue staining

Aniline blue staining of callose was performed as described by Conrath *et al.* (1998). Leaves were placed in a phenol solution (water: glycerine: phenol: lactic acid, 1:2:2:1 mass mixture), boiled for 2 min, and then subjected to three 5 min washes with distilled water. The clarified leaves were then dyed with 0.01% aniline blue solution (in 0.1 M PBS; pH 7.0) for 15 min and subsequently washed three times (5 min each) with distilled water, after which they were imaged under an ultraviolet excitation filter (BP 330–385 nm) via a fluorescence microscope (BX50, Olympus, Japan). The experiment was performed three times. The turquoise fluorescence was regarded manually as the regions of interest (ROI) for quantification. Callose deposition was quantified by turquoise fluorescence of callose deposits using ImageJ 2x software. The ‘Freehand selections’ or ‘Magic wand’ was used to define the area of a callose deposit, and ‘Histogram list’ as the reference for calculating fluorescence intensity (Ellinger *et al.*, 2013). Average callose measurements were based on at least six photographs from different leaves.

### Western blot assays

The total protein of leaves was extracted with a Plant Protein Extraction Kit (Solarbio, Beijing, China) and quantified via a BCA Protein Assay Kit (Beyotime, Shanghai, China). Following this, 40 µg of protein was loaded onto 10% SDS-PAGE gels; following electrophoresis the proteins were transferred to polyvinylidene fluoride (PVDF) membranes (Millipore, USA), and the membranes were blocked with 5% skimmed milk powder at 4 °C for 3 h. The membranes were subsequently incubated together with anti- β -1,3-glucanase antibodies (AS07-208; 1:200, Agrisera, Sweden) and anti-plant Rubisco antibodies (QYA00813A, 1:2000; Beijing Qualityard, Beijing, China) overnight at 4 °C, after which they were incubated together with HRP-conjugated goat anti-mouse IgG (QYB001; 1:5000; Beijing Qualityard, Beijing, China) or HRP-conjugated goat anti-rabbit IgG (QYB002; 1:5000; Beijing Qualityard, Beijing, China) for 2 h at 4 °C. The protein fragments were observed with an enhanced chemiluminescence (ECL) kit (Millipore, USA) under a chemiluminescence imaging system (ChemiDoc XRS+, Bio-Rad, USA). The fragments were analysed and quantified via ImageJ 2x software.

### Transmission electron microscopy immunolocalization

The leaves were cut into 1×3 mm pieces that were then placed in a fixation solution consisting of 4% paraformaldehyde and 0.1% glutaraldehyde for 2 h at 4 °C. The samples were then subjected to three 10 min washes with 0.1 M PBS, after which they were dehydrated using different concentrations of ethanol (30% for 30 min, 50% for 30 min, 70% for 1 h, 90% for 1 h, 100% for 3 h, changing the 100% ethanol every hour) at 4 °C. The leaves were subjected to three additional 10 min washes with 0.1 M PBS, permeated with ethanol: K4 M resin (1:3) for 1 h and then ethanol: K4 M resin (1:1) for 3 h, embedded in pure K4 M resin for 24 h at 4 °C, polymerized at –20 °C for 72 h, and then kept at 25 °C for 48 h in a UV polymerization box (Electron Microscopy China, Beijing, China). The embedded mass was sliced into semi-thin sections to observe the location, after which 90 nm ultrathin sections were cut with an ultramicrotome (EM UC7, Leica, Germany) and collected on formvar-coated Au grids (Electron Microscopy China, Beijing, China). The sections were subjected to three 5 min washes with 0.1 M PBS and double-distilled water, and then blocked in 1% bovine serum albumin (BSA) at 25 °C for 30 min. The sections were incubated together with anti-β-1,3-glucan antibodies (1:100; G7527; Biosupplies, Parkville, Australia) or anti-β-1,3-glucanase antibodies (1:50; AS07-208; Agrisera, Sweden) at 37 °C for 2 h, and then subjected to three 5 min washes with 0.1 M PBS and double distilled water. The sections were subsequently incubated together with anti-mouse IgG/Gold conjugated to 5 nm colloidal gold (1:100; G7527; Sigma, USA) or goat anti-rabbit IgG/Gold conjugated to 10 nm colloidal gold (1:100; bs-0295G-Gold; Bioss, Beijing, China) at 37 °C for 1 h. The sections were subjected to three 5 min washes with 0.1 M PB and double distilled water, and subsequently stained with 2% (w/v) uranyl acetate for 5 min, followed by lead citrate for 5 min at 25 °C. The sections were ultimately observed and imaged with a transmission electron microscope (JEM-1400PLUS, JEOL, Japan).

### Ultrastructural observations

Leaves were subjected to three 10 min washes with 0.1 M PBS and then fixed at 4 °C in 1% (w/v) osmium tetroxide for 3 h. Following this, the samples were subjected to three 10 min washes with 0.1 M PBS, dehydrated via different concentrations of ethanol, permeabilized and embedded in Epon812 resin (SPI, USA), which was then polymerized at 60 °C. The embedded mass was sliced into semi-thin sections to observe the location, and ultrathin sections were then cut with an ultramicrotome (EM UC7, Leica, Germany), after which they were collected onto formvar-coated Cu grids (Electron Microscopy China, Beijing, China). The sections were stained with 2% (w/v) uranyl acetate for 15 min, followed by lead citrate for an additional 7 min. The sections were then observed and imaged with a transmission electron microscope (TECNAI G2, FEI, USA). The plasmodesma diameters were measured at the neck region via the TEM Imaging & Analysis software (FEI, USA; Schindelin *et al.*, 2012; Liesche *et al.*, 2019).

### Statistical analysis

Statistical significance was determined by Student’s *t*-tests or one-way ANOVA via GraphPad Prism version 5.0a (GraphPad Software). The experiments were performed at least three times.

## Results

### Identification of interesting DEGs

To investigate the potential mechanism underlying the PDMP-induced resistance of *N. benthamiana* in response to TMV, we focused on the PDMP/TMV versus TMV comparison; detailed information on the DEGs identified from this comparison is shown in Table 1. As shown in Table 1, three genes were annotated; then, we focused on the interesting DEGs (*IRAK4* (*interleukin-1 receptor-associated kinase4*), *ABA2* and *β-1,3-glucanase*). The expression of interesting genes identified from all the comparisons are shown in Table 2. The results indicated that the expression of *β-1,3-glucanase* and *IRAK4* was up-regulated in the TMV versus control and PDMP/TMV versus control comparisons, but down-regulated in the PDMP/TMV versus TMV comparison. We also found that the expression of *ABA2* was down-regulated in the TMV versus control comparison and up-regulated in the PDMP/TMV versus TMV comparison; however, no difference in the expression of these genes was observed in the PDMP versus control comparison (Table 2). These results suggest that compared with TMV inoculation alone, PDMP pre-treatment before TMV inoculation could suppress the expression of *β-1,3-glucanase* and promote the expression of *ABA2*.

**Table 1.** Detailed information on the DEGs in the PDMP/TMV versus TMV comparison.

Gene ID	Gene description	KEGG annotation	Log_2_FC	*P* value	Regulation
Niben101Scf17372g01008	Probable LRR receptor-like serine/threonine-protein kinase	IRAK4	-2.007887	1.6E-26	down
Niben101Scf02839g02012	-	-	-1.330981	4.9E-25	down
Niben101Scf11624g00001	-	-	1.31642	1.4E-12	up
Niben101Scf04641g00012	Secoisolariciresinol dehydrogenase	ABA2	1.217216	1.9E-12	up
Niben101Scf04641g01003	Secoisolariciresinol dehydrogenase	ABA2	1.070924	4.7E-11	up
Niben101Scf27377g00004	-	-	1.142838	1.7E-09	up
Niben101Scf02175g06018	-	-	-1.137547	9.2E-09	down
Niben101Scf01001g00005	β-1,3-glucanase	-	-1.032814	2.6E-08	down
Niben101Scf03814g00003	-	-	-1.031907	2.3E-07	down
Niben101Scf04309g02001	-	-	1.019251	3.2E-07	up

**Table 2.** Expression of the interesting genes identified in all comparisons.

Gene ID	Gene description	KO annotation	PDMP versus control	TMV versus control	PDMP/TMversus control	PDMP/TM versus TMV
Niben101Scf17372g01008	Probable LRR receptor-like serine/threonine-protein kinase	IRAK4	N	up	up	down
Niben101Scf04641g00012	Secoisolariciresinol dehydrogenase	ABA2	N	down	N	up
Niben101Scf04641g01003	Secoisolariciresinol dehydrogenase	ABA2	N	down	N	up
Niben101Scf01001g00005	β-1,3-glucanase	-	N	up	up	down

N: no significant difference (*P*>0.05). Statistical significance was determined by one-way ANOVA.

Because β-1,3-glucanase is associated with callose deposition, and ABA2 is involved in ABA biosynthesis, we speculated that PDMP inhibited TMV movement by inducing callose priming; moreover, the ABA biosynthetic pathway might play an important role in PDMP-induced resistance to TMV.

### Validation of DEGs

**Fig. 1.**
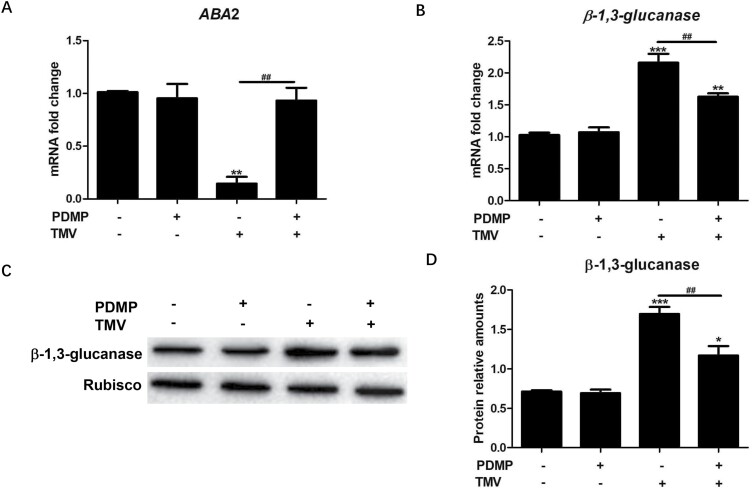
qRT–PCR-based validation of interesting DEGs (*ABA*2 and *β-1,3-glucanase*). The mRNA expression of *ABA*2 (A) and *β-1,3-glucanase* (B) were detected via qRT–PCR assay. The cycle threshold (CT) values of the target genes were normalized to the CT value of *β-actin*. (C) β-1,3-glucanase protein was detected by western blot assay. (D) Quantitative results of β-1,3-glucanase protein fragments. Protein relative amounts of β-1,3-glucanase were quantified relative to the expression of Rubisco. Values are means ±SD (*n*=6). *: *P*<0.05, **: *P*<0.01 and ***: *P*<0.001 versus the control treatment; ^##^: *P*<0.001, versus TMV treatment, as determined by one-way ANOVA.

qRT–PCR assays were performed to validate the expression of interesting DEGs (*β-1,3-glucanase* and *ABA2*). The results indicated that the expression trends of both DEGs revealed by qRT–PCR were in accordance with those revealed by RNA-seq (Fig. 1; Supplementary Fig. S1), which suggests that the RNA-seq data reliably reflected the gene expression changes.

As shown in Fig. 1A, *ABA2* expression significantly decreased (*P*<0.01) in the TMV treatment compared with that in the control treatment, PDMP reversed the TMV-induced decrease in *ABA2* expression in the PDMP/TMV treatment, and no significant difference (*P*>0.05) in *ABA2* expression was found among the PDMP, PDMP/TMV, and control treatments. These results indicated that ABA biosynthesis might be associated with PDMP-induced defence. As shown in Fig. 1B-D, *β-1,3-glucanase* mRNA and protein expression significantly increased (*P*<0.001) in the TMV treatment compared with those in the control treatment. Moreover, PDMP decreased *β-1,3-glucanase* expression in the PDMP/TMV treatment compared with that in the TMV treatment; however, *β-1,3-glucanase* expression in the PDMP/TMV treatment was still greater compared with the control. β-1,3-glucanase is involved in callose deposition; thus, we speculated that PDMP might induce callose deposition in response to TMV infection. Moreover, the role ABA plays in PDMP-induced callose deposition as a defence response against TMV has not been clarified.

### Successful generation of a *N. benthamiana ZEP* mutant

**Fig. 2.**
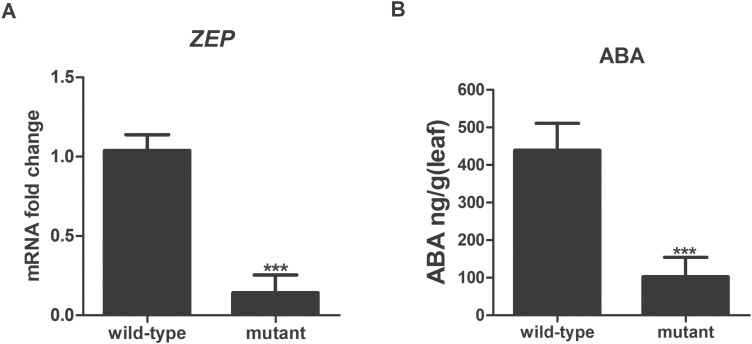
Validation of CRISPR/gRNA-mediated *NbZEP* genetic mutants. (A) The expression of *ZEP* was detected via qRT–PCR assays. The cycle threshold (CT) values of the target genes were normalized to the CT value of *β-actin*. (B) ABA concentration in the leaves of the *NbZEP* mutant and wild-type plants was determined by an ELISA. Values are means ±SD (*n*=20). ***: *P*<0.001 versus wild-type, as determined by Student’s *t* test.

The *ZEP* gene encodes an enzyme that plays a catalytic role in the intermediate reaction of the ABA biosynthetic pathway. We first cloned the *ZEP* gene of *N. benthamiana*; the nucleic acid and amino acid sequences of *NbZEP* are shown in Supplementary Fig. S2A. To investigate whether the ABA biosynthetic pathway participates in PDMP-induced callose priming, we generated an *N. benthamiana ZEP* mutant via *Agrobacterium*-mediated infection in conjunction with CRISPR/Cas9 gene-editing technology. The target sequence and mutated bases are shown in Supplementary Fig. S2B. In the mutant plants, a single-base insertion of G was detected in the exon of *NbZEP*. The insertion, which was located at position 74 in *NbZEP*, produced a premature stop codon (TAA) in the open reading frame (ORF), resulting in a loss-of-function mutation (Supplementary Fig. S2). The mRNA expression of *NbZEP* in T2 *ZEP* mutants was measured via a qRT–PCR assay (Fig. 2A), and the ABA concentration in the T2 *ZEP* mutants was measured via an ELISA (Fig. 2B). As shown in Fig. 2, the expression of *NbZEP* and the ABA concentration in the mutants were markedly inhibited compared with the wild-type. The results suggested that a *N. benthamiana* ABA biosynthesis mutant had been generated successfully.

### Movement of TMV-GFP and callose deposition

**Fig. 3.**
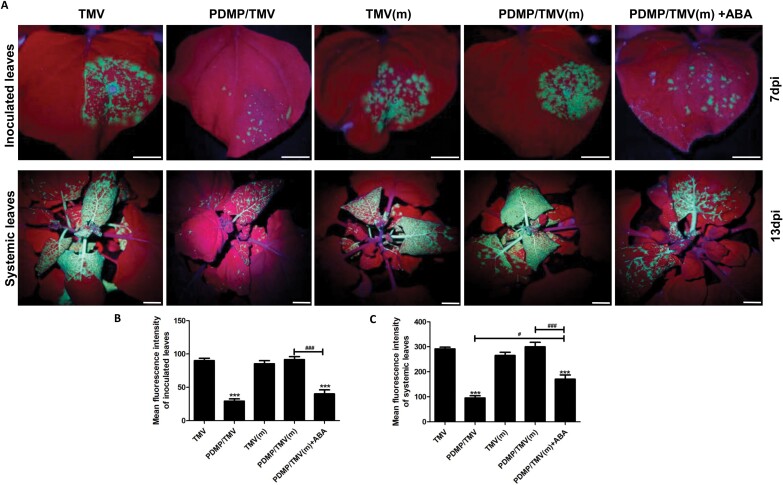
Presence of GFP-TMV movement. (A) Movement of TMV is shown by green fluorescence under a UV lamp. Scale bar =1 cm. The green fluorescence in the inoculated leaves (B) and systemic leaves (C) was quantified via ImageJ software. Values are means ±SD (*n*=6). ***: *P*<0.001 versus TMV treatment; ^#^: *P*<0.05 and ^###^: *P*<0.001, as determined by one-way ANOVA. ‘m’ indicates ABA biosynthesis mutant.

To investigate the effects of PDMP on resistance to TMV infection, we observed the movement of GFP-labelled TMV with the naked eye under a handheld UV lamp, and detected callose deposition in *N. benthamiana* by aniline blue staining and microscopy. As shown in Fig. 3A, GFP fluorescence was detected in the inoculated leaves (7 dpi) and the systemic leaves (13 dpi) of both wild-type and mutant plants in the TMV group and the PDMP/TMV group. Quantitation of the green fluorescence of the inoculated and systemic leaves is shown in Fig. 3B, C, respectively. In the wild-type plants, the fluorescence intensity of GFP-labelled TMV in the inoculated and systemic leaves in the PDMP/TMV treatment was markedly reduced compared with that in the TMV treatment alone. In the mutant plants, no difference was found between the TMV and PDMP/TMV treatments. Treatment with exogenous ABA significantly decreased (*P*<0.001) the fluorescence intensity of TMV in the inoculated and systemic leaves of the mutant plants (Fig. 3B, C). These results suggest that PDMP can significantly suppress the speed of TMV movement, and that its inhibitory effect on TMV could be rescued in the mutant by treatment with exogenous ABA.

**Fig. 4.**
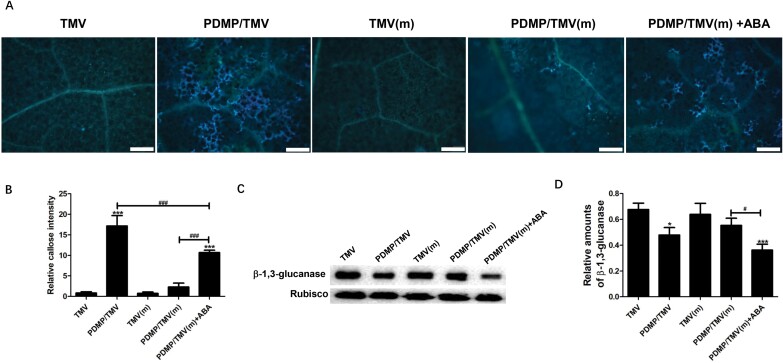
Presence of callose fluorescence and callose-related protein (β-1,3-glucanase). (A) Callose deposition is shown as turquoise fluorescence after staining with aniline blue and imaged under an ultraviolet excitation filter (BP 330–385 nm) via a fluorescence microscope. Scale bar =100 µ m. (B) Relative callose intensities emitted by aniline blue-stained callose deposits (*n*=6). The turquoise fluorescence was regarded as the regions of interest (ROI). The turquoise fluorescence was chosen and quantified manually using ImageJ software. (C) Western blot validation of β-1,3-glucanase expression. (D) Protein fragments were quantified relative to the expression of Rubisco via ImageJ software (*n*=3). Values are means ±SD. *: *P*<0.05 and ***: *P*<0.001 versus TMV treatment; ^#^: *P*<0.05 and ^###^: *P*<0.001, as determined by one-way ANOVA. ‘m’ indicates ABA biosynthesis mutant.

As shown in Fig. 4A, B, callose deposition was observed as turquoise fluorescence after staining with aniline blue and imaged under a fluorescence microscope. These results suggested that PDMP significantly (*P*<0.001) promoted callose deposition in the wild-type plants inoculated with TMV, whereas little callose deposition was observed in the PDMP/TMV treatment in the mutant. No significant difference (*P*>0.05) was found between the TMV and PDMP/TMV treatments in the mutant. However, significantly increased (*P*<0.001) callose deposition was found in the PDMP/TMV treatment of the mutant with ABA compared with the PDMP/TMV treatment of the mutant (Fig. 4A, B). In addition, western blotting showed that in the wild-type plants, β-1,3-glucanase protein amounts in the PDMP/TMV treatment were lower than those in the TMV treatment, whereas no significant difference (*P*>0.05) was found between the TMV treatment and PDMP/TMV treatment in the mutant. However, treatment with ABA decreased the amount of β-1,3-glucanase protein in the mutant plants (Fig. 4C, D). Taken together, these results suggested that PDMP-induced callose deposition priming depended on ABA biosynthesis.

### Immunolocalization of callose

**Fig. 5.**
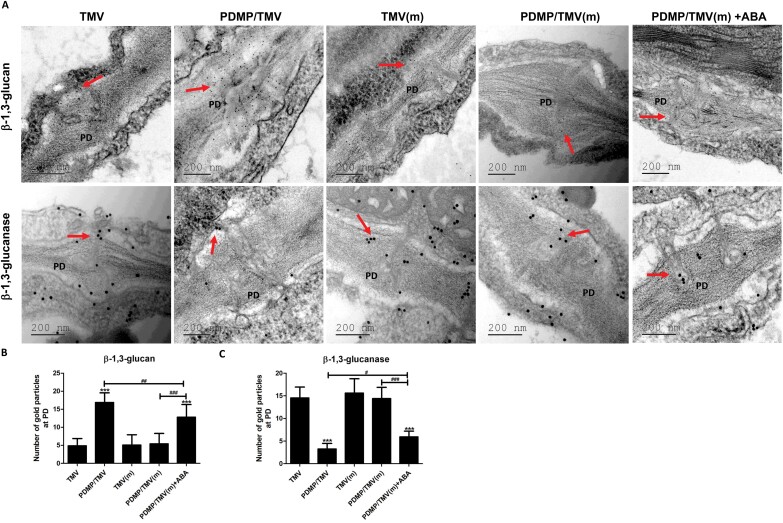
Analysis of immunocolloidal gold coupled with transmission electron microscopy of callose deposits around plasmodesmata. (A) β -1,3-glucan and β -1,3-glucanase were detected around plasmodesmata in the different treatments. Scale bar =200 nm. Quantitative results of β -1,3-glucan (B) and β -1,3-glucanase (C) gold particles around plasmodesmata. The number of gold particles was counted per individual plasmodesma (8–10 plasmodesmata were analysed per sample). Values are means ± SD (*n*=3). ***: *P*<0.001 versus TMV treatment; ^#^: *P*<0.05, ^##^: *P*<0.01 and ^###^: *P*<0.001, as determined by one-way ANOVA. PD: plasmodesma. The red arrows indicate callose particles. ‘m’ indicates ABA biosynthesis mutant.

To directly confirm the induction of callose deposition around plasmodesmata by PDMP, β-1,3-glucan and β-1,3-glucanase proteins were detected through immunolocalization experiments. Consistent with the results from the aniline blue staining experiments, compared with the TMV treatment, PDMP/TMV treatment of wild-type plants presented greater amounts of immunogold-labelled β-1,3-glucan and lower amounts of β-1,3-glucanase (Fig. 5A) around the plasmodesmata. However, there was no significant difference (*P*>0.5) in immunogold-labelled β-1,3-glucan or β-1,3-glucanase between the TMV and the PDMP/TMV treatments in the mutant (Fig. 5A-C). Compared with the TMV-treated wild-type plants, the PDMP/TMV treated wild-type plants presented a greater number of β-1,3-glucan gold particles around the plasmodesmata, but a lower number of β-1,3-glucanase gold particles, whereas no significant difference (*P*>0.05) in the number of β-1,3-glucan or β-1,3-glucanase gold particles was found between the TMV treatment and the PDMP/TMV treatment in the mutant. However, the addition of ABA increased the number of β-1,3-glucan gold particles and decreased that of β-1,3-glucanase gold particles. Thus, these results indicate that in *N. benthamiana*, PDMP induces the deposition of callose at plasmodesmata to stimulate resistance to TMV infection through the ABA biosynthetic pathway.

### Ultrastructure and diameters of plasmodesmata

**Fig. 6.**
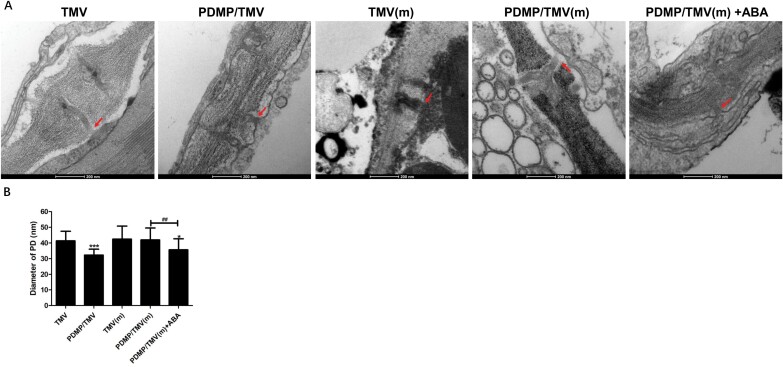
Plasmodesmata structural parameters. (A) Plasmodesmata ultrastructure was observed via transmission electron microscopy. (B) Plasmodesmata diameters (8–10 plasmodesma were analysed per sample) were measured at the neck region via the TEM Imaging & Analysis software. Scale bar =200 nm. Values are means ±SD (*n*=27). *: *P*<0.05 and ***: *P*<0.001 versus TMV treatment; ^##^: *P*<0.01, as determined by one-way ANOVA. The red arrows show the neck of plasmodesmata. The letter ‘m’ indicates ABA biosynthesis mutant.

To further verify the results from the aniline blue staining and immunolocalization experiments, the ultrastructure of plasmodesmata were observed by transmission electron microscopy (Fig. 6A). Moreover, the plasmodesmata diameters were measured, and the results are shown in Fig. 6B. These results showed that for the wild-type plants, the PDMP/TMV treatment resulted in narrower plasmodesmata than the TMV treatment; moreover, no significant difference (*P*>0.5) was found between the TMV and PDMP/TMV treatments in the mutant. Treatment with ABA along with PDMP/TMV decreased the plasmodesmata diameter in the mutant. These results were similar to those obtained from both the aniline blue labelling and immunolocalization experiments. Taken together, the results demonstrated that in *N. benthamiana*, PDMP induced the deposition of callose around the plasmodesmata in cell walls to stimulate resistance to TMV infection through the ABA biosynthetic pathway.

## Discussion

TMV is one of the main viruses that harm tobacco plants in many countries (Zhao *et al.*, 2017), causing economic losses in commercial crops through its infection of host plants; however, safe and effective prevention and treatment methods for TMV are lacking. Researchers have focused on the study of fungal extracts that can protect tobacco from TMV, because a variety of proteins, polysaccharides, and other substances that exert strong anti-TMV activity have been found in various fungi (Chen and Gao, 2006; Li *et al.*, 2014). Few studies have investigated PDMP-mediated resistance to TMV and the underlying mechanism, although DMP has been reported to induce resistance in several plant species under field conditions (Chen *et al.*, 2006; Dong *et al.*, 2006).

In this study, RNA-seq technology was first used to investigate the comprehensive resistance mechanisms induced by PDMP with TMV infection in *N. benthamiana*. We focused on the differential expression of genes between the PDMP/TMV versus TMV treatments, and found interesting DEGs (*β-1,3-glucanase* and *ABA2*). The results indicated that *β-1,3-glucanase* and *ABA2* were not differentially expressed in the PDMP versus control comparison, but were differentially expressed in the PDMP/TMV versus TMV comparison (Table 2), which revealed that PDMP induces priming to resist TMV infection. Despite the limitations of the analysis involving RNA-seq data (many inaccurate or imprecise classifications for GO term analysis), our RNA-seq data implied that PDMP induces priming to promote resistance to TMV infection. Some studies have demonstrated that BABA (β -aminobutyric acid)-induced tolerance to osmotic stress is based on priming to achieve enhanced adaptation responses rather than the direct activation of these responses (Hasegawa *et al.*, 2000; Jakab *et al.*, 2005). In our study, pre-treatment with PDMP did not induce a direct response, but rather induced defence responses after TMV challenge, which indicated that PDMP can induce priming to defend against TMV infection and help tobacco leaves rapidly establish a resistance response after TMV challenge. However, the specific signalling pathways involved in PDMP-induced priming are unclear.

ABA can induce plant defence against viruses (Chen *et al.*, 2013; Alazem and Lin, 2015), and ABA-related recessive resistance against two RNA viruses (bamboo mosaic virus and cucumber mosaic virus) has been reported (Alazem *et al.*, 2014). β-1,3-glucanase, an enzyme that hydrolyses β-1,3-glycosidic bonds in β-1,3-glucans, has been proposed to degrade cell walls (Leubner-Metzger and Meins, 1999). Moreover, *β-1,3-glucanase* expression was down-regulated and *ABA2* expression was up-regulated in the PDMP/TMV versus TMV comparison, which indicates that callose deposition and the ABA pathway might participate in the PDMP-induced priming of resistance to TMV. Although other hormones may also be involved in the regulation of callose (DebRoy *et al.*, 2004; Pieterse *et al.*, 2009; Vicedo *et al.*, 2009) and thus in resistance to TMV, we focused on ABA in this study.

Higher plants indirectly synthesize ABA through a series of catalytic reactions, in which many important enzymes are involved (Fraser and Bramley, 2004; Breitenbach and Sandmann, 2005). Moreover, ZEP, NCED (9-*cis*-epoxycarotenoid dioxygenase), and AO aldehyde oxidase) play important roles in this pathway. It has been reported that in *Nicotiana plumbaginifolia aba2* mutants, ABA synthesis is hindered by attenuated oxidation of zeaxanthin, and the enzyme that catalyses epoxidation is *ZEP*, which is encoded by the tobacco *ABA*2 gene (Marin *et al.*, 1996). Heterologous expression of the alfalfa zeaxanthin epoxidase gene (*MsZEP*) can confer drought and salt tolerance in *Nicotiana tabacum* (Zhang *et al.*, 2016). These results revealed that *ZEP* plays important roles in ABA biosynthesis. Moreover, it has been reported that the pre-treatment of tobacco with ABA contributes to increased resistance against TMV (Fraser, 1982). ABA can down-regulate β-1,3-glucanase activity in tobacco cell cultures (Rezzonico *et al.*, 1998); thus, we speculated that PDMP-induced callose priming for resistance against TMV might depend on the ABA biosynthetic pathway.

To test our hypothesis, *N. benthamiana ZEP* mutants were successfully obtained using the CRISPR/Cas9 technique. We found that, compared with the PDMP/TMV treatment, the PDMP/TMV treatment in the mutant clearly exhibited faster TMV movement and less callose deposition, which indicated that PDMP cannot induce callose deposition to defend against TMV in the *ZEP* mutants. However, whether factors other than callose deposition are involved in the restriction of virus movement cannot be excluded. Nevertheless, exogenous ABA treatment could rescue the inhibitory effect of PDMP on TMV. PDMP induces callose deposition through a mechanism dependent on ABA biosynthesis. A previous study indicated that ABA promotes callose deposition and that overexpression of the ABA biosynthesis gene *NCED3* reduces *PR2* expression and increases callose deposition in *Arabidopsis thaliana* (Oide *et al.*, 2013). Also, exogenous ABA suppresses β-1,3-glucanase activity and induces synthase activity, both of which are beneficial for the promotion of callose deposition in rice (Liu *et al.*, 2017). Our results led to similar conclusions concerning the positive effect of ABA biosynthesis on callose deposition.

Plant hormone signalling pathways are crucial for plant immune responses and callose deposition. Salicylic acid (SA) is involved in microbe-triggered callose deposition (DebRoy *et al.*, 2004). Moreover, both jasmonic acid (JA)-signal priming and callose deposition are mechanisms involved in hexanoic acid-induced resistance against *Botrytis cinerea* in tomato plants (Vicedo *et al.*, 2009). The plant defence system ultimately consists of crosstalk among signalling pathways and different hormones (Pieterse *et al.*, 2009). In the present study, we focused on the effects of ABA on PDMP-induced resistance against TMV in tobacco plants. We describe a link among ABA, callose degradation, and callose deposition in PDMP-induced defence priming. The successful infection of a plant by a virus depends on cell-to-cell movement through plasmodesmata (Lucas, 1995; Ding, 1998; Lucas and Lee, 2004), and the modification of plasmodesmata by callose deposition could restrict the movement of viruses between cells (Chen and Kim, 2009; Epel, 2009; Hofmann *et al.*, 2010). The defence-related gene *OCP3* (overexpressor of cationic peroxidase 3) represents a specific control point for callose deposition regulated by jasmonic acid, but ultimately requires ABA (García-Andrade *et al.*, 2011). We have demonstrated that PDMP could be considered a priming stimulus and that ABA positively regulates callose deposition upon challenge with TMV. Whether other plant hormones (salicylic acid, jasmonic acid) interplay with ABA to regulate the resistance induced by PDMP needs further work.

To further investigate whether the ABA biosynthetic pathway plays a crucial role in PDMP-induced callose deposition around plasmodesmata to defend against TMV movement, the immunolocalization of callose-related proteins was detected via immunocolloidal gold assays. Moreover, ultrastructure and diameters of plasmodesmata were also observed and detected. Callose is an effective barrier against virus movement, and callose deposition is negatively correlated with β-1,3-glucanase (a callose-degrading enzyme) content in tobacco during resistance to TMV (Beffa *et al.*, 1996). Our results suggest that the PDMP-induced callose deposition around plasmodesmata and impaired TMV movement depend on the ABA biosynthetic pathway. Moreover, exogenous ABA treatment rescued the promoting effect of PDMP on callose deposition around plasmodesmata. Thus, the ABA biosynthetic pathway may directly or indirectly influence callose deposition around plasmodesmata. In our study, the results suggested that the ABA biosynthetic pathway suppresses β-1,3-glucanase activity to trigger changes in callose deposition around plasmodesmata during PDMP-induced resistance to TMV. Moreover, ABA may indirectly promote callose deposition around plasmodesmata via other regulatory mechanisms, such as the regulation of lipid raft-related proteins (Huang *et al.*, 2019) and the promotion of starch degradation (Gamir *et al.*, 2018). The indirect effects of ABA on callose during PDMP-induced resistance need further studies. The intact indole-3-carboxylic acid (I3CA) biosynthetic pathway has been reported to be necessary for I3CA-induced resistance (Pastor-Fernández *et al.*, 2019). I3CA can induce resistance in Arabidopsis against the necrotrophic fungus *Plectosphaerella cucumerina* through priming callose accumulation (Gamir *et al.*, 2018; Pastor-Fernández *et al.*, 2019). In our study, we found that the ABA biosynthetic pathway is necessary for PDMP-induced resistance; however, the underlying mechanism needs to be studied further in depth.

Our study also revealed the interesting DEG Niben101Scf17372g01008, which is annotated as a ‘probable LRR receptor-like serine/threonine-protein kinase’; this gene may be associated with TMV or PDMP binding. Many studies have revealed that the leucine-rich repeat receptor-like kinase (LRR-RLK) BAK1 is a major modulator of PTI in *Arabidopsis thaliana* (Chinchilla *et al.*, 2007; Heese *et al.*, 2007). The receptor-like kinase BAK1 is crucial for *N. benthamiana* resistance to pathogens (Chaparro-Garcia *et al.*, 2011). Therefore, we are interested in investigating whether LRR-RLK interacts with PDMP and is related to TMV infection.

As demonstrated in our previous study, PDMP markedly activates the defence of *N. glutinosa* against TMV and restricts the spread of TMV (Zhong *et al.*, 2015). In the present study, our results revealed for the first time that PDMP-induced callose priming in *N. benthamiana* against TMV was dependent on the ABA biosynthetic pathway. These findings could provide a theoretical foundation for the application of PDMP as a biocontrol agent. Although further research is needed to assess any additional factors involved in PDMP-induced resistance to TMV in tobacco, the present data show that PDMP-induced resistance is based on priming callose deposition and on the enhanced ABA biosynthetic pathway, which contribute to the knowledge of PDMP-induced resistance of tobacco plants.

## Supplementary data

The following supplementary data are available at *JXB* online.

Fig. S1. qRT-PCR-based validation of genes (*ABA*2, *β-1,3-glucanase*, *ZEP*).

Fig. S2. CRISPR/gRNA-mediated construction of *NbZEP* genetic mutant.

Table. S1. Primer sets used in this study.

erab102_suppl_Supplementary_Figures_S1_S2_and_Table_S1

## Data Availability

The data supporting the findings of this study are available from the corresponding author (JW), upon request.

## Abbreviations

ABA: abscisic acid
CRISPR/Cas9: clustered regularly interspaced short palindromic repeats/CRISPR-associated protein 9
DEG: differentially expressed gene
DMP: dry mycelium of *Penicillium chrysogenum*
NCED: 9-*cis*-epoxycarotenoid dioxygenase
GFP: green fluorescent protein
PAM: protospacer adjacent motif
PDMP: polypeptide extract of dry mycelium of *Penicillium chrysogenum*
TMV: tobacco mosaic virus
ZEP: zeaxanthin epoxidase
