# Supplementary material for: Penicillium chrysogenum polypeptide extract protects tobacco plants from tobacco mosaic virus infection through modulation of ABA biosynthesis and callose priming
Source: J Exp Bot. 2021 Mar 3;72(10):3526–39. doi: 10.1093/jxb/erab102 (PMC8096601; doi:10.1093/jxb/erab102)
Supplement: erab102_suppl_Supplementary_Figures_S1_S2_and_Table_S1 [file erab102_suppl_supplementary_figures_s1_s2_and_table_s1.pdf]

Supporting Information

Fig. S1 qRT-PCR-based validation of genes (*ABA*2, *β-1,3-glucanase*, *ZEP*). The cycle threshold (CT) values of the target genes were normalized to the CT value of *EF1α* (A) and *F-box* (B). The data are presented as the means ± standard deviations (n=6). **: $p < 0.01$ and ***: $p < 0.001$ vs the control group or the wild-type group; #: $p < 0.05$, ##: $p < 0.01$ and ###: $p < 0.001$, vs the TMV group, as determined by one-way ANOVA.

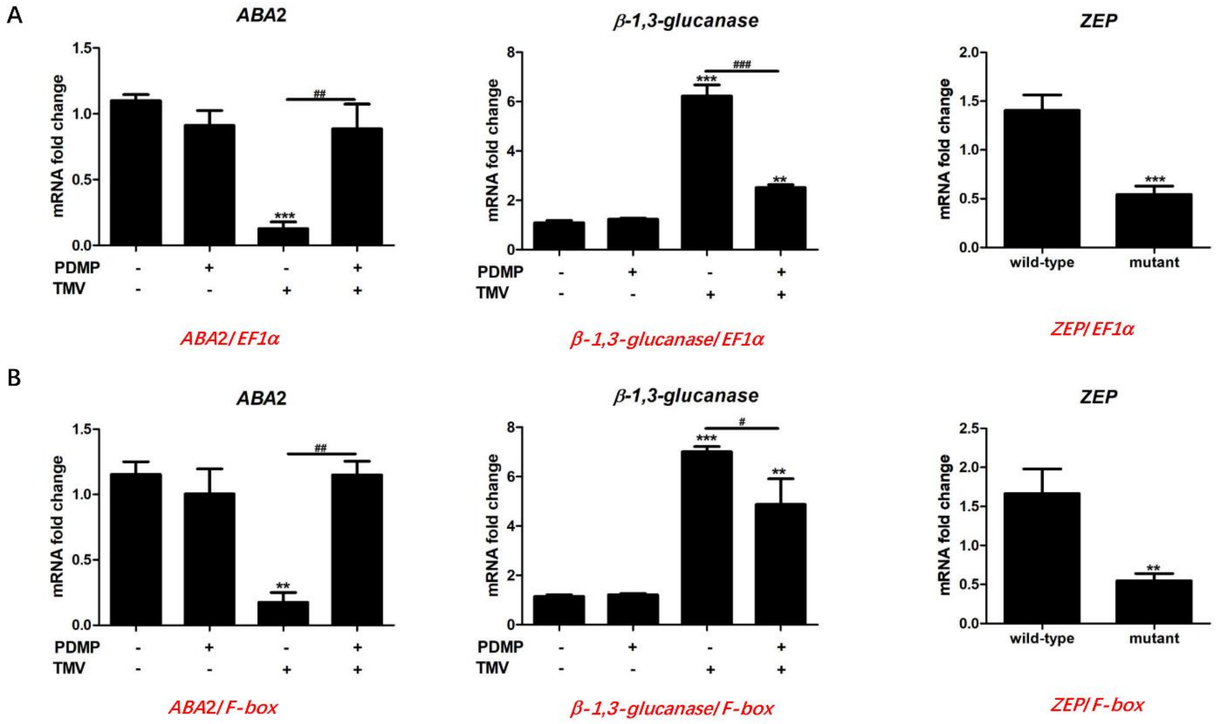

Fig. S2 CRISPR/gRNA-mediated *NbZEP* genetic mutant construction. (A) Base sequences and amino acid sequences of *NbZEP*. (B) Sequences of the mutated sites in homozygous *NbZEP* mutant plants and schematic representation of the mutated position in *NbZEP* mutant plants. Insertions are indicated by red letters, and the numbers on the right side indicate the size of the insertions. The inserted bases are shown in red letters, and premature stop codons are shown in the red box.

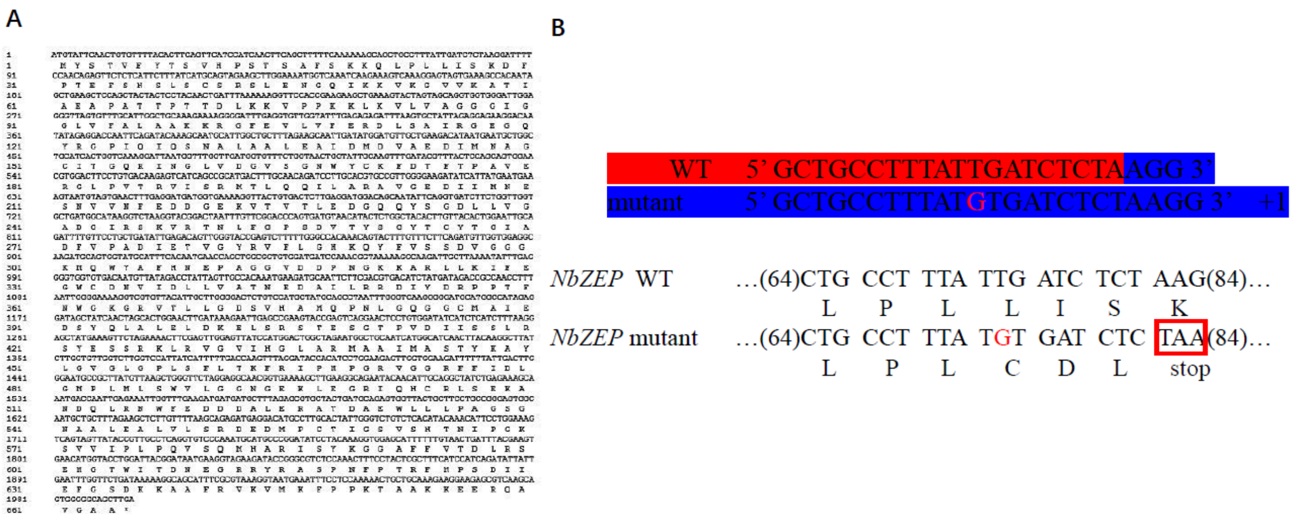

Table. S1 Primer sets used in this study.

Target gene	Sequence (5’-3’)
β-ACTIN	AACTGGGACGATATGGAGAA
	CACTGGCGTATAGGGACAAC
EF1α	CCCAAGAGGCCCTCAGACA
	CACACGACCAACAGGGACAGT
F-BOX	GGCACTCACAAACGTCTATTTC
	ACCTGGGAGGCATCCTGCTTAT
ZEP	GTGGAACGTGGACTTCCTGT
	TACATCACTGGGTCCGAACA
ABA2	AGTAGCAGGTGGTGGGATTG
	TCCATGTCAATTGCTTCCAA
β-1,3-GLUCANASE	ATACCTACCCGCCCAAAGAT
	TCCAAAAGGGCATCAAAAAG
